# Osteoporosis knowledge and awareness in hemiplegic patients: a cross-sectional study

**DOI:** 10.1590/1806-9282.20252274

**Published:** 2026-08-24

**Authors:** Elif Ozyigit, Mert Zure

**Affiliations:** 1 University of Health Sciences, Kanuni Sultan Suleyman Training and Research Hospital, Department of Physical Medicine and Rehabilitation – Istanbul, Turkey.

**Keywords:** Hemiplegia, Osteoporosis, Health knowledge, attitudes, practice, Educational status, Cognition

## Abstract

**OBJECTIVE:**

Osteoporosis is a prevalent but often overlooked condition in hemiplegic patients that increases the risk of fracture. The aim of this study was to evaluate osteoporosis knowledge and awareness levels and determine associated factors in adult hemiplegic individuals.

**METHODS:**

A cross-sectional study was conducted with 88 hemiplegic participants (45 males, 43 females) in a tertiary rehabilitation clinic. Demographic and clinical data were documented. Cognitive function was assessed using the Mini-Mental State Examination, while osteoporosis knowledge was evaluated through the 31-item Osteoporosis Awareness Scale in addition to a structured question ("Have you ever heard of osteoporosis?"). Statistical analyses included independent t-tests, one-way analysis of variance with Tukey post-hoc testing, Pearson correlations, and multiple linear regression (significance: p<0.05).

**RESULTS:**

The mean Osteoporosis Awareness Scale score was 72.55±32.29 (range: 32–124). Seventy-two (81.8%) participants reported having heard of osteoporosis. In univariate analysis, Osteoporosis Awareness Scale scores were higher in females (76.58±33.81 vs. 68.73±30.52, p=0.036), postmenopausal women (78.92±33.45 vs. 58.40±26.31, p=0.042), those with higher education levels (p<0.001), and individuals with better cognitive function (p<0.001). Multiple linear regression confirmed that education level (p<0.001) and Mini-Mental State Examination score (p=0.002) were significant independent predictors of Osteoporosis Awareness Scale scores. Gender (p=0.052) and menopausal status (p=0.087) were not significant in the multivariate model.

**CONCLUSION:**

Education level and cognitive function were significant independent predictors of osteoporosis knowledge in hemiplegic patients. Gender and menopausal status were not significant after adjustment for education and cognitive function. These findings highlight the need for targeted educational strategies in rehabilitation settings, particularly for patients with lower educational backgrounds and cognitive impairment.

## INTRODUCTION

Osteoporosis (OP) is a common yet often underestimated health concern among individuals with hemiplegia. Characterized by decreased bone mineral density and deterioration of bone microarchitecture, OP significantly increases fracture risk, which can lead to severe morbidity, disability, and even mortality^1^. It is particularly relevant in hemiplegic patients, a population in which bone health is substantially affected. Despite this importance, OP is frequently underdiagnosed and undertreated^2^.

Hemiplegia, typically resulting from stroke or other brain injuries, significantly impairs physical function, limiting mobility and weight-bearing activities. This immobility contributes to accelerated bone loss, particularly in the paralyzed limbs^2,3^. The increased fracture risk due to impairments in balance, gait, and coordination, especially at sites like the hip, represents a major concern given its association with prolonged recovery times and higher mortality rates^3,4^.

Patient knowledge is pivotal in preventing OP and its complications. However, OP is often called the "silent disease" as symptoms may not appear until significant bone loss has occurred. Studies reveal widespread lack of awareness of OP and its risk factors among patients and healthcare providers^5–7^. Research in community settings shows knowledge gaps and limited use of risk assessment tools^5,8^. In hemiplegia, a knowledge deficit is even more concerning, as early diagnosis and preventive strategies are crucial for mitigating fracture risk^3,4^.

Healthcare professionals have a critical responsibility to educate hemiplegic patients about OP. Effective patient education should include information on risks, preventive strategies, the benefits of weight-bearing exercise, nutrition, and bone density screening. Systemic barriers, such as limited access to diagnostic tools and educational resources, must also be addressed^5,7–9^.

Although studies have examined OP awareness in various patient populations, evidence regarding OP knowledge among individuals with hemiplegia remains scarce. Moreover, to the best of our knowledge, no previous study has specifically evaluated OP awareness in a hemiplegic population using a validated OP awareness scale. Given the increased risk of OP and fractures in these patients, understanding their level of knowledge is essential for developing effective educational and preventive strategies. Therefore, this study aimed to assess OP knowledge among adult hemiplegic patients and identify factors associated with awareness levels, thereby providing evidence to guide targeted rehabilitation-based educational interventions.

## METHODS

### Study design and setting

Between February 2023 and March 2024, a cross-sectional study was conducted among hemiplegic patients attending a tertiary rehabilitation clinic. Two hundred patients were screened. Inclusion criteria were diagnosis of hemiplegia, age ≥18 years, and voluntary participation. Exclusion criteria included prior OP diagnosis, Mini-Mental State Examination (MMSE) score <24, and inability to complete the questionnaire due to severe communication impairments. Of the 200 participants screened, 112 were excluded: 58 had MMSE scores <24, 28 had prior OP diagnosis, 18 refused to participate, and 8 were unable to complete the questionnaire due to severe communication impairments. The final sample consisted of 88 participants (45 males and, 43 females).

A post-hoc power analysis using G*Power 3.1 indicated that with 88 participants and four predictors in the multivariate model, the study had 95% power to detect a medium effect size (f^2^=0.15) at α=0.05.

### Assessments and data collection

Demographic data (age, gender, body mass index [BMI], education level, occupation, and marital status) and clinical data (comorbidities, corticosteroid use, fracture history, menopausal status, duration of hemiplegia) were collected through structured interviews and medical record review.

Cognitive function was assessed using the MMSE^10^. OP knowledge was evaluated using two approaches: (1) a structured yes/no question asking "Have you ever heard of osteoporosis?" and (2) the validated Osteoporosis Awareness Scale (OAS)^11^. The OAS is a 31-item instrument using a four-point Likert scale (1–4), with total scores ranging from 31 to 124; higher scores indicate greater knowledge about OP. For clarity, the term "osteoporosis" was replaced with "bone loss" in the Turkish version^11^.

### Ethical considerations

Ethical approval was obtained from the Clinical Research Ethics Committee of Kanuni Sultan Süleyman Training and Research Hospital (decision no: 4, date: 11.01.2023). Written informed consent was obtained from all participants, and the study adhered to the Declaration of Helsinki.

### Statistical analysis

Statistical Package for the Social Sciences 25.0 was used for all analyses. Data normality was evaluated with the Shapiro-Wilk test and histograms. Continuous variables were compared using independent samples t-test or Mann-Whitney U test depending on distribution. Categorical variables were compared using the chi-square test. Pearson correlation coefficients were calculated to assess relationships between OAS scores and continuous variables (age, BMI, MMSE score, duration of hemiplegia). One-way analysis of variance (ANOVA) with Tukey post-hoc testing was used to compare OAS scores across education levels. Multiple linear regression analysis was performed to identify independent predictors of OAS scores. Variables with p<0.10 in univariate analyses were entered into the multivariate model. A p<0.05 was considered statistically significant.

## RESULTS

**Figure 1:**
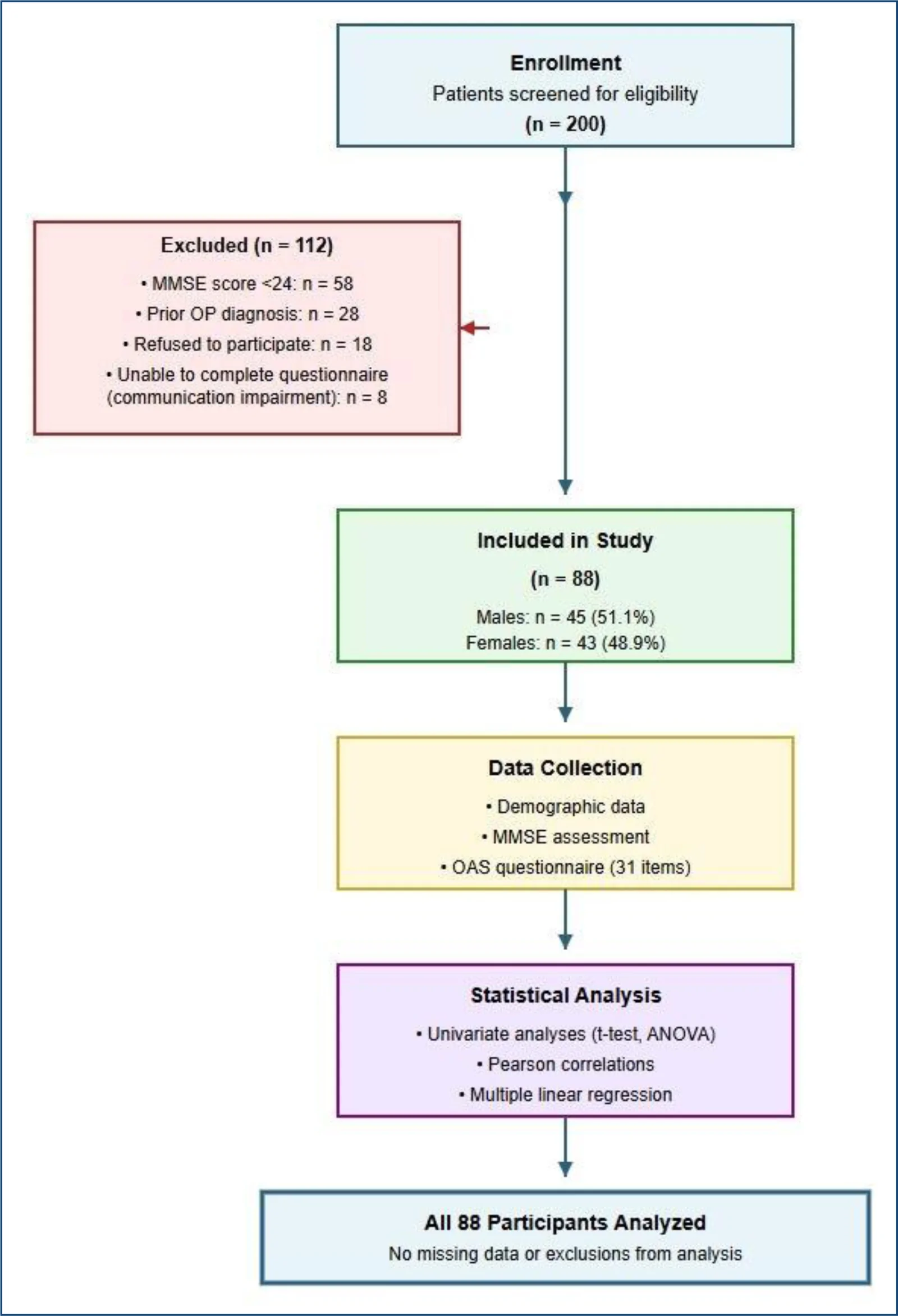
Study flowchart.

Of 200 screened patients, 88 met the inclusion criteria and participated in the study (Figure 1). Of the participants, 45 were male (51.1%) and 43 female (48.9%). Mean age was 64.1±14.7 years; mean duration since hemiplegia onset was 64.5±24.3 months. Mean BMI was 27.3±3.8 kg/m^2^. Educational levels varied, with 12 participants (13.6%) holding university degrees and 12 (13.6%) being illiterate. When asked "Have you ever heard of osteoporosis?," 72 patients (81.8%) responded "yes" while 16 (18.2%) responded "no." Demographic and clinical characteristics are presented in Table 1.

**Table 1:** Demographic and clinical characteristics of hemiplegic patients and comparison of Osteoporosis Awareness Scale scores.

Variable		Total (n=88)	Mean OAS score±SD	p-value
Gender				
	Male	45 (51.1%)	68.73±30.52	0.036*
	Female	43 (48.9%)	76.58±33.81	
Age (years), mean±SD		64.1±14.7	r=-0.089	0.408
BMI (kg/m^2^), mean±SD		27.3±3.8	r=0.053	0.625
Duration of hemiplegia (months), mean±SD		64.5±24.3	r=-0.121	0.262
Education level				
	Illiterate	12 (13.6%)	45.75±10.46	<0.001** †
	Primary school	28 (31.8%)	58.21±18.35	
	Middle school	19 (21.6%)	72.89±22.41	
	High school	17 (19.3%)	85.53±27.16	
	University	12 (13.6%)	112.67±26.85	
Menopausal status (females only, n=43)				
	Premenopausal	5 (11.6%)	58.40±26.31	0.042* ‡
	Postmenopausal	38 (88.4%)	78.92±33.45	
MMSE score, mean±SD		27.45±1.52	r=0.486§	<0.001**
Heard of osteoporosis				
	Yes	72 (81.8%)	78.93±31.05	<0.001**
	No	16 (18.2%)	46.38±17.87	
Comorbidities				
	Present	75 (85.2%)	73.21±32.84	0.587
	Absent	13 (14.8%)	68.85±29.12	
Corticosteroid use				
	Yes	8 (9.1%)	69.25±28.73	0.724
	No	80 (90.9%)	72.91±32.78	
Fracture history				
	Yes	22 (25.0%)	68.18±30.45	0.391
	No	66 (75.0%)	74.02±32.91	

OAS: Osteoporosis Awareness Scale; SD: standard deviation; BMI: body mass index; MMSE: Mini-Mental State Examination.

* <0.05;

** p<0.

† One-way analysis of variance with Tukey post-hoc test.

‡ Small sample size of premenopausal women (n=5) limits interpretation.

§ Pearson correlation coefficient.

The mean OAS score for the entire sample was 72.55±32.29, with a range of 32–124. Female participants had higher mean OAS scores compared to males (76.58±33.81 vs. 68.73±30.52, p=0.036; mean difference 7.85, 95%CI 0.56–15.14). Among the 43 female participants, 38 were postmenopausal, and five were premenopausal. Postmenopausal women had higher OAS scores compared to premenopausal women (78.92±33.45 vs. 58.40±26.31, p=0.042; mean difference 20.52, 95%CI 0.79–40.25). However, given the small sample size of premenopausal women (n=5), this finding should be interpreted with caution.

Education level showed a strong positive correlation with OAS scores (Pearson r=0.520, p<0.001). Participants with university education had the highest mean OAS scores (112.67±26.85), while illiterate participants had the lowest (45.75±10.46). One-way ANOVA confirmed significant differences across education levels (F=18.42, p<0.001), with Tukey post-hoc testing showing significant pairwise differences between most groups. Given the multiple comparisons in Table 1 (13 tests), we acknowledge the increased risk of Type I error. As this was an exploratory study, we did not apply a formal Bonferroni correction; however, borderline p-values (0.036 and 0.042) should be interpreted with caution.

MMSE scores demonstrated a significant positive correlation with OAS scores (Pearson r=0.486, p<0.001). Participants who responded "yes" to having heard of OP had significantly higher mean OAS scores (78.93±31.05 vs. 46.38±17.87, p<0.001; mean difference 32.55, 95%CI 18.42–46.68).

Age showed no significant correlation with OAS scores (r=-0.089, p=0.408). The most common comorbidities were hypertension (64.8%), diabetes mellitus (42.0%), and coronary artery disease (30.7%). No significant differences in OAS scores were found based on the presence of comorbidities (p=0.587), corticosteroid use (p=0.724), or history of fracture (p=0.391). The non-significant findings for corticosteroid use (n=8, 9.1%) and fracture history (n=22, 25%) may reflect Type II error due to small subgroup sizes or may indicate that these factors genuinely do not influence knowledge in this population. Duration of hemiplegia and BMI also showed no significant associations with OAS scores.

Multiple linear regression analysis (Table 2) was performed with the OAS score as the dependent variable. Variables included in the model were gender, education level, menopausal status (for females), and MMSE score. The model was statistically significant (F=18.24, p<0.001, R^2^=0.479, adjusted R^2^=0.448) and explained 44.8% of the variance in OAS scores. Education level (unstandardized B=10.87, standardized β=0.389, p<0.001, 95%CI 6.09–15.65) and MMSE score (unstandardized B=6.74, standardized β=0.312, p=0.002, 95%CI 2.61–10.87) emerged as significant independent predictors. Gender (p=0.052) and menopausal status (p=0.087) were not significant in the multivariate model.

**Table 2:** Multiple linear regression analysis of factors associated with Osteoporosis Awareness Scale scores in hemiplegic patients.

Variable	Unstandardized B (SE)	Standardized β	95%CI	p-value
Constant	−115.42 (45.82)	—	−206.25 to −24.59	0.013
Gender (female)	11.24 (5.68)	0.176	−0.05 to 22.53	0.052
Education level	10.87 (2.41)	0.389	6.09 to 15.65	<0.001**
Menopausal status (postmenopausal)	14.32 (8.25)	0.165	−2.08 to 30.72	0.087
MMSE score	6.74 (2.08)	0.312	2.61 to 10.87	0.002**

SE: standard error; CI: confidence interval; MMSE: Mini-Mental State Examination; NS: not significant. Model: F=18.24, p<0.001, R^2^=0.479, adjusted R^2^=0.448.

** p<0.01.

## DISCUSSION

This study examined OP knowledge in hemiplegic patients using the validated OAS and identified education level and cognitive function as the primary determinants of knowledge. The mean OAS score of 72.55±32.29 indicates moderate overall knowledge, though considerable variability existed across participants (range: 32–124). While 81.8% of participants had heard of OP, recognition alone does not equate to comprehensive knowledge about the condition, its risk factors, or preventive measures.

Education level emerged as the strongest independent predictor of OAS scores (β=0.389, p<0.001). This finding is consistent with previous studies showing that higher education correlates with greater OP knowledge across diverse populations^7–9,12–15^. University-educated participants scored more than twice as high as illiterate participants (112.67 vs. 45.75), highlighting the need for educational interventions tailored to varying literacy levels. Individuals with higher education may possess better general literacy and numeracy skills that facilitate understanding of health information, greater self-efficacy in seeking and processing health information, more frequent healthcare interactions, and better access to educational resources^9,13,14^.

Cognitive function, as measured by MMSE, was also a significant predictor (β=0.312, p=0.002), underscoring the importance of cognitive capacity in health literacy. The relationship between cognitive function and health knowledge has theoretical support from cognitive load theory and health literacy frameworks, which posit that adequate working memory, attention, and executive function are necessary for processing and retaining health information^15,16^. Our exclusion criterion of MMSE <24 ensured participants could meaningfully engage with the questionnaire, but this threshold may have excluded the most cognitively impaired individuals who might benefit most from simplified educational approaches.

In univariate analysis, female gender and postmenopausal status were associated with higher OAS scores (p=0.036 and p=0.042, respectively). However, these variables were not significant in the multivariate model after adjustment for education and cognitive function (p=0.052 and p=0.087, respectively). These findings suggest that while women may have greater engagement with bone health issues, the effect is modest and may be confounded by other factors such as educational attainment and healthcare utilization patterns. Importantly, the small sample size of premenopausal women (n=5) limits the reliability of comparisons by menopausal status.

Contrary to some prior reports^17–19^, fracture history, comorbidities, and corticosteroid use were not significantly associated with OAS scores. This may reflect Type II error due to small subgroup sizes (e.g., only eight patients used corticosteroids) or may indicate that hemiplegic patients do not fully appreciate the link between their condition and bone health. This represents a missed opportunity for patient education in clinical settings.

Age showed no significant correlation with OAS scores, suggesting that chronological age alone does not determine knowledge levels and that educational efforts should not assume older patients are necessarily more or less informed.

These findings have important clinical implications for rehabilitation practice. Educational interventions should particularly target hemiplegic patients with lower cognitive function and lower educational attainment, who appear to be at greater risk of inadequate OP knowledge. Materials should be developed at multiple literacy levels, using visual aids and simplified language for those with limited education. In addition, structured OP awareness programs may be incorporated into routine rehabilitation services, allowing healthcare professionals to provide education on bone health, fracture prevention, nutrition, physical activity, and the importance of OP screening. Such approaches may improve health literacy, encourage preventive behaviors, and ultimately contribute to reducing fracture risk in this vulnerable population^5,15,20,21^. Health literacy principles provide a framework for developing accessible educational materials, including using plain language (sixth-grade reading level), visual aids, limiting messages to 3–4 key points, and incorporating 40–50% white space^22^.

This study has several limitations. First of all, the cross-sectional design precludes causal inference. Second, the relatively small sample from a single center limits generalizability. Patients with prior OP diagnosis were excluded, which may have biased the sample toward those with lower knowledge and potentially underestimated the true range of OP awareness. Multiple comparisons were performed without formal correction, increasing the risk of Type I error; borderline p-values (0.036, 0.042) should be interpreted with caution. The small sample size for certain subgroups (e.g., corticosteroid users [n=8], premenopausal women [n=5]) may have resulted in Type II error for non-significant findings. Including both gender and menopausal status in the same model may have introduced collinearity. In addition, only knowledge levels were assessed, not actual bone mineral density or clinical outcomes.

Future studies should test the effectiveness of structured educational programs tailored to different literacy levels and conduct longitudinal follow-ups to assess knowledge retention and its impact on clinical outcomes, including bone mineral density and fracture incidence.

## CONCLUSION

Education level and cognitive function were significant independent predictors of OP knowledge in hemiplegic patients. Gender and menopausal status were not significant after adjustment for these factors. The wide variability in knowledge scores highlights the need for individualized educational strategies in rehabilitation settings, particularly for patients with lower educational backgrounds and cognitive impairment. Future studies should evaluate whether enhanced educational programs lead to improved knowledge, behavior change, and ultimately, reduced fracture incidence in this high-risk population.

## Data Availability

The datasets generated and/or analyzed during the current study are available from the corresponding author upon reasonable request.
